# A H-REV107 Peptide Inhibits Tumor Growth and Interacts Directly with Oncogenic KRAS Mutants

**DOI:** 10.3390/cancers12061412

**Published:** 2020-05-30

**Authors:** Chang Woo Han, Mi Suk Jeong, Sung Chul Ha, Se Bok Jang

**Affiliations:** 1 Department of Molecular Biology, College of Natural Sciences, Pusan National University, 2, Busandaehak-ro 63 beon-gil, Geumjeong-gu, Busan 46241, Korea; hotorses@pusan.ac.kr; 2 Korea Nanobiotechnology Center, Pusan National University, 2, Busandaehak-ro 63 beon-gil, Geumjeong-gu, Busan 46241, Korea; 3 Pohang Accelarator Laboratory, Pohang University of Science and Technology, 80 Jigok-ro 127 beon-gil, Nam-gu, Gyeongsangbuk-do, Pohang-si 37673, Korea; scha2@postech.ac.kr

**Keywords:** KRAS, H-REV107, oncogenic mutation, tumor suppressor

## Abstract

Kirsten-RAS (KRAS) has been the target of drugs because it is the most mutated gene in human cancers. Because of the low affinity of drugs for KRAS mutations, it was difficult to target these tumor genes directly. We found a direct interaction between KRAS G12V and tumor suppressor novel H-REV107 peptide with high binding affinity. We report the first crystal structure of an oncogenic mutant, KRAS G12V-H-REV107. This peptide was shown to interact with KRAS G12V in the guanosine diphosphate (GDP)-bound inactive state and to form a stable complex, blocking the activation function of KRAS. We showed that the peptide acted as an inhibitor of mutant KRAS targets by [α-^32^P] guanosine triphosphate (GTP) binding assay. The H-REV107 peptide inhibited pancreatic cancer and colon cancer cell lines in cell proliferation assay. Specially, the H-REV107 peptide can suppress pancreatic tumor growth by reduction of tumor volume and weight in xenotransplantation mouse models. Overall, the results presented herein will facilitate development of novel drugs for inhibition of KRAS mutations in cancer patients.

## 1. Introduction

RAS was identified by the extensive study of retroviral oncogenes isolated from the genome rat-derived Harvey and Kirsten murine sarcoma viruses [1]. RAS is a proto-oncogene that is mutated in human cancer, and the RAS protein is encoded by three expressed genes: Harvey-Ras (HRAS), Kirsten-RAS (KRAS), and neuroblastoma-Ras (NRAS) [2]. The H^−^, K^−^, and NRas proteins are small GTPases that serve as master regulators of countless signaling cascades involved in particularly diverse cellular processes, such as cell division, differentiation, cell–cell adhesion, growth, and apoptosis. The small GTPase RAS proteins operate as “molecular switches” that fluctuate between inactive and active states. These switches can be activated by the exchange of guanosine triphosphate (GTP) for guanosine diphosphate (GDP), which is promoted by guanine nucleotide exchange factors (GEFs). In contrast, they are inactivated when GTP is hydrolyzed to GDP, which is promoted by GTPase-activating proteins (GAPs) [3].

Activating mutations in RAS are found in certain human cancers. Some 9–30% of human tumors have RAS-activating mutations, which are common in KRAS (86%), NRAS (11%), and HRAS (3%) [4,5]. KRAS has been a target for drug design for more than 30 years because it is the most commonly mutated oncogene in human cancers, including pancreatic (90%), colon (40%), and non-small cell lung cancers (20%) [6,7]. The National Cancer Institute (NCI) recently highlighted the importance of this drug goal to bring together researchers who are developing new ideas to stop Ras by announcing a USD 10 million effort called the RAS project. The project is designed to promote the development of new drugs or treatment that can benefit cancer patients.

Point mutations in the *KRAS* gene are present in over 90% of pancreatic ductal adenocarcinoma (PDAC) cases and are thought to be an early event in the development of PDAC that is already occurring in PanIN 1A lesions of the pancreas [5,8]. Kulemann et al. compared KRAS mutations in pancreatic circulating tumor cells (CTC) and corresponding tumors, and evaluated their significance as prognostic markers [9]. Cancers with RAS mutations are aggressive and respond poorly to standard therapies; accordingly, they have earned a reputation as being “undruggable” because scientific researchers have failed to design a drug that successfully targets the mutant gene. These mutations render RAS proteins insensitive to GTP-induced hydrolysis of GTP to GDP and lock them in the activated state. Carcinogenic mutations cause functional activation of Ras family proteins by impairing GTP hydrolysis [6]. Thus far, potential inhibition molecules have been reported to almost indirectly target RAS-functional interactions without binding to RAS.

The KRAS mutations are usually found to affect residue G12 and less commonly residues G13 and Q61 [4,5]. For example, the most common KRAS mutation types are G12C, G12D, and G12V accounting for almost 83% of all KRAS mutations [10]. Recently, Liu’s group showed that a covalent inhibitor specific for G12C mutant KRAS induces tumor regression in in vivo models [11]. *KRAS* mutations in ovarian serous borderline tumors (OSBTs) and ovarian low-grade serous carcinomas (LGSCs) have previously been reported [12,13]. Interestingly, ovarian carcinoma patients with the KRAS *G12V* mutation appeared to have shorter overall survival than those without this mutation [14]. For these reasons, selectively targeting the KRAS G12V mutant is among the highest priorities of ovarian cancer therapy. The number of mice developing lymph node metastases was also found to be higher in KRas G12V (73%) and KRas G13D (29%) than in KRas wild-type (11%) mice. KRas G12V showed higher tumor cell survival, invasion, and CXCR4 expressing intravasated tumor emboli than KRas G13D. In human CRC tumors, of 12 different point mutations found at KRas codon 12 or 13, only the KRas G12V mutation conveyed an increased risk of recurrence and death [15]. The mutation at codon 13 has been found to occur predominantly in a subset of CRCs defective in DNA repair genes, while Q61 is essential for GTP hydrolysis and its mutation blocks Ras-mediated GTP hydrolysis and leads to tumor formation [16,17,18].

The H-REV107 gene is a known member of the class II tumor suppressor gene family that has been identified as a growth inhibitory RAS target capable of suppressing anchorage independent growth in vitro an in vivo [19]. Several studies have shown that H-REV107 is ubiquitously expressed in most normal tissues, however, expression is lost in human tumor cell lines and tumor samples. Furthermore, H-REV107 expression is strongly reduced in approximately 50% of human ovarian carcinomas. Loss of the human H-REV107 in ovarian carcinoma cells is a result of reversible down-regulation via the MAP/ERK pathway. In contrast, induction of H-REV107 expression resulted in growth inhibition of RAS-transformed cells in vitro and in vivo [20,21,22,23]. We previously modeled the binding of H-REV107 protein to GNP-bound KRAS mutation (Q61H) [24].

Because of the very low affinity of the drug for KRAS mutations, it was difficult to target these tumor genes directly. Here, we found direct interaction and inhibition between KRAS G12V and novel H-REV107 peptide. It acts as an inhibitor of mutant KRAS targets (G12V, G12D, G12C, G13D, and Q61H) by [α-^32^P] GTP binding assay. Treatment with the H-REV107 peptide effectively inhibited pancreatic cancer and colon cancer cell lines in cell proliferation assay by inducing apoptosis. We also determined the first crystal structure of an oncogenic mutant—KRAS G12V protein bound with novel H-REV107 peptide. The peptide directly interacts with KRAS G12V at the GDP-bound inactive state and peptide bound KRAS G12V forms a stable open form complex that can block RAS activation function. Specifically, the peptide can suppress pancreatic tumor growth by reduction of tumor volume and weight in xenotransplantation mouse models. Overall, the results of this study will facilitate development of new effective drugs for the inhibition of KRAS mutations in patients with cancer.

## 2. Results

### 2.1. The H-REV107 Peptide Bound to KRAS G12V with High Binding Affinity

**Figure 1:**
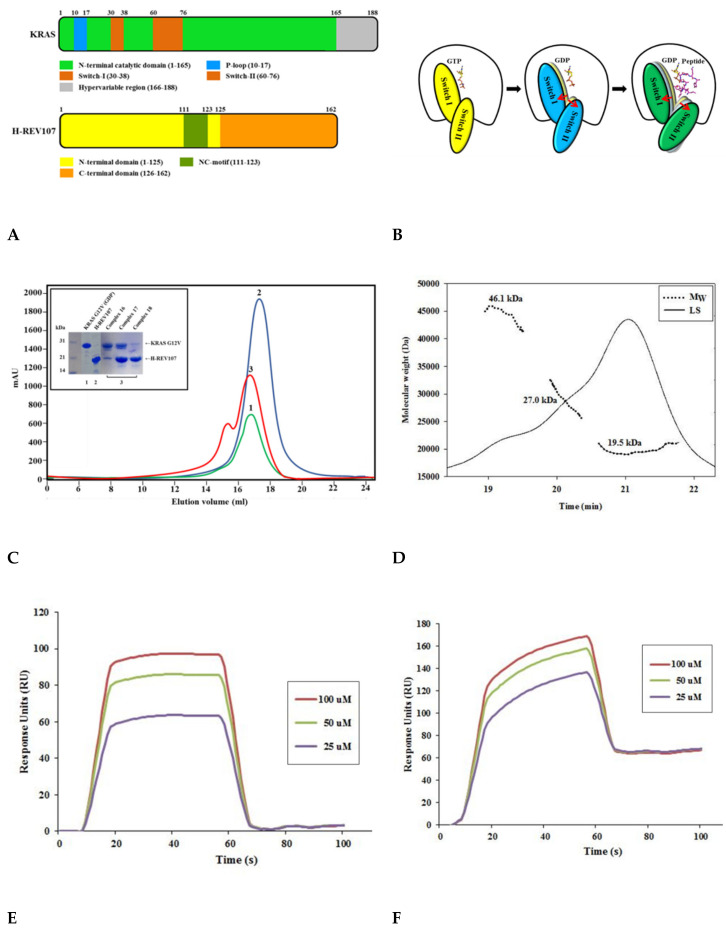
Schematic representation and biophysical properties of KRAS and H-REV107. (**A**) Domain structures of full-length KRAS and H-REV107 are shown. (**B**) Cartoon model of KRAS in closed (left: guanosine triphosphate (GTP)-bound), opened (middle: guanosine diphosphate (GDP)-bound), and further opened (right: GDP- and peptide-bound) forms is shown. (**C**) Size-exclusion chromatography of KRAS G12V and H-REV107 proteins. The interaction band (red) of KRAS G12V (green) with H-REV107 (blue) after application of size exclusion chromatography from the Superdex 200 column was analyzed by SDS-PAGE. (**D**) Size-exclusion chromatography–multi-angle light scattering (SEC-MALS) spectrum of the KRAS and H-REV107 complex. The inset shows the value of the molecular weight of KRAS and H-REV107 determined from the MALS data analysis (black line: MALS, dashed line: molecular weight). (**E**,**F**) Biacore biosensor analysis of KRAS mutant (G12V and G12D) binding to H-REV107 peptide at 25 °C. H-REV107 peptide sensorgrams for 25, 50, and 100 µM are shown.

First, we expressed and purified KRAS G12V and H-REV107 to understand their molecular interactions (Figure 1A–C and Figure S1A,B). The oncogenic KRAS mutants (G12V, G12D, G12C, G13D, and Q61H) were then constructed and purified, after which their secondary structures were studied. The circular dichroism (CD) spectra of the KRAS mutants showed that each mutation affected the conformation of KRAS to a different extent. Size-exclusion chromatography (SEC) analysis revealed the presence of KRAS G12V and H-REV107 complex in the final purified peak (Figure 1C). Using SEC-MALS (multi-angle light scattering), we found that the KRAS G12V and H-REV107 complex had a molecular mass of 46 kDa on the SDS-PAGE gel. The band of His-KRAS G12V (amino acids 1–188) was located at 27 kDa, while that of His-H-REV107 (aa 1–125) was at 19 kDa. The peak of the KRAS G12V-H-REV107 complex was shown as the sum of the molecular mass of a 1:1 complex (Figure 1D). The complex of the KRAS G12V and H-REV107 protein was purified; however, the complex crystal was not obtained. Nevertheless, we successfully obtained the crystal of KRAS G12V and H-REV107 peptide complex (Figure S2E). The H-REV107 peptide is highly soluble in water. The binding affinity of the H-REV107 protein/peptide to KRAS G12V was determined by Biacore biosensor analysis. In our previous work, the K_D_ values of WT or Q61H KRAS were determined by surface plasmon resonance (SPR) to be 1–9 nM [24]. These were shown high binding affinities with the H-REV107 protein in the previous study. In this paper, the H-REV107 peptide also showed high binding affinities to G12V or G12D, with a K_D_ of 1–3 μM. By using isothermal titration calorimetry (ITC) analysis, KRAS G13D, Q61H, and G12C showed binding affinities to the peptides, with K_D_ = 17–50 μM. KRAS G12V showed binding affinity to H-REV107 protein with K_D_ = 30 μM. The ITC results in Figure S3A,B showed ambiguous binding affinities (Figure 1E,F and Figure S3).

### 2.2. The GTP Binding to the KRAS Mutants Was Greatly Decreased in the Presence of H-REV107 Peptide

**Figure 2:**
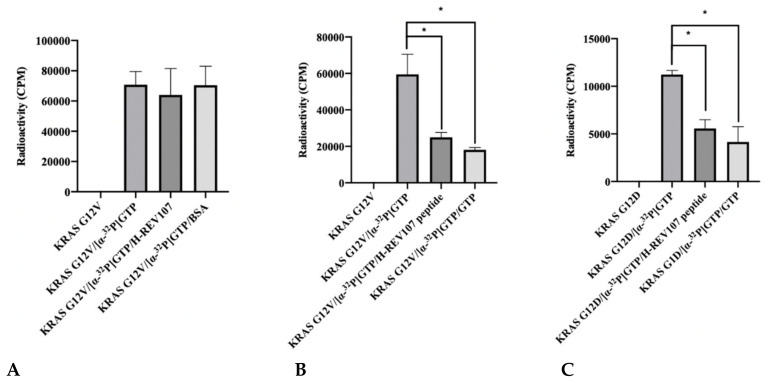

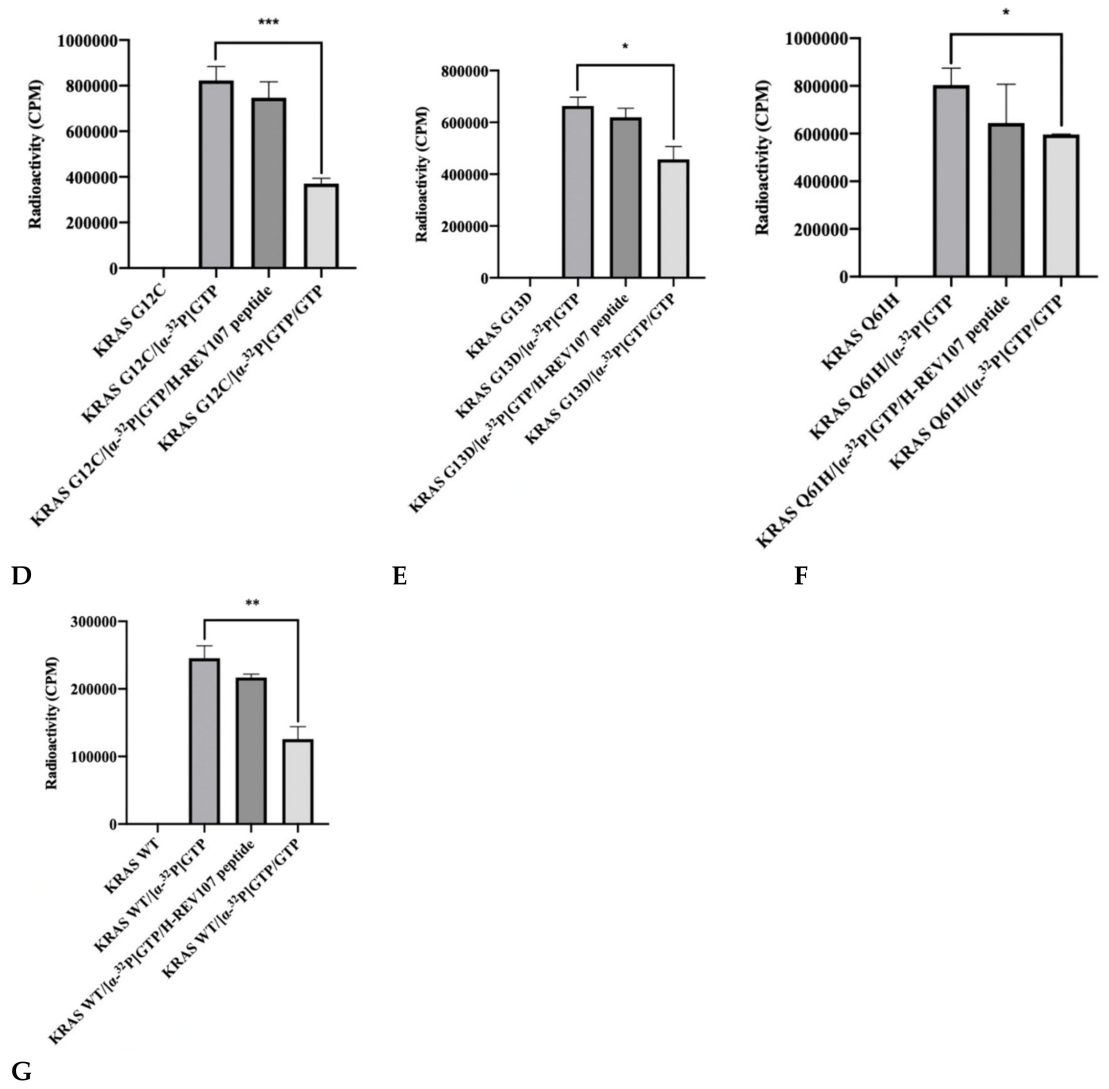
Inhibitor assay of KRAS mutants and H-REV107 protein/peptide. (**A**) The binding of GTP to KRAS mutant (G12V) in the presence of H-REV107 protein was determined using α^32^-labeled GTP. (**B**–**G**) The binding of GTP to KRAS mutants (G12V, G12D, G12C, G13D, and Q61H) or wild-type in the presence of H-REV107 peptide was determined using α^32^-labeled GTP. The non-bound ^32^α-GTP was washed out and the radioactivity was examined using a scintillation counter. Non-labeled GTP was used as a competitor. Data are presented as mean ± SD. Statistic tests were performed using a Student’s *t*-test. * *p* < 0.05, ** *p* < 0.01, and *** *p* < 0.001 compared to the control under α^32^-labeled GTP (second column).

The reduction of GTP binding affinity to KRAS mutant was predicted to be linked to the interaction with KRAS oncogenic mutants (G12V, G12D, G12C, G13D, and Q61H) and H-REV107. We evaluated this hypothesis by [α-^32^P] GTP binding assay using KRAS mutant and H-REV107 protein with bovine serum albumin (BSA) as a negative control. The addition of KRAS G12V and BSA both resulted in high levels of [α-^32^P] GTP binding in the absence of H-REV107 protein. However, the [α-^32^P] GTP binding activity of KRAS G12V was decreased (9%) in the presence of H-REV107 protein (Figure 2A). We also evaluated the binding ability of H-REV107 peptide in a [α-^32^P] GTP binding assay using KRAS mutants (G12V, G12D, G12C, G13D, and Q61H) with unlabeled guanosine 5^’^-tirphosphate (GTP) as a negative control. The results showed that [α-^32^P] GTP binding to the KRAS mutants was greatly decreased in the presence of H-REV107 peptide (Figure 2B–G). Following the addition of H-REV107 peptide, GTP binding to the KRAS mutants (G12V, G12D, and G12C) decreased to 50%, 40%, and 10%, respectively, of the GTP binding activities to KRAS mutants. In addition, the GTP binding activities to KRAS Q61H, G13D, and wild-type were decreased to 20%, 7%, and 12%, respectively. These findings indicate that the H-REV107 peptide (^65^LYDVAGSDKY^74^) inhibits the interaction between KRAS mutant targets (G12V, G12D, G12C, G13D, and Q61H) and GTP. Among their interactions, GTP binding to the KRAS G12V mutant by the H-REV107 peptide showed the greatest decrease (Figure 2B), indicating that the H-REV107 peptide inhibits the oncogenic mutant KRAS G12V well.

### 2.3. Inhibitory Effect of H-REV107 Peptide Was Observed on Tumor Cells

**Figure 3:**
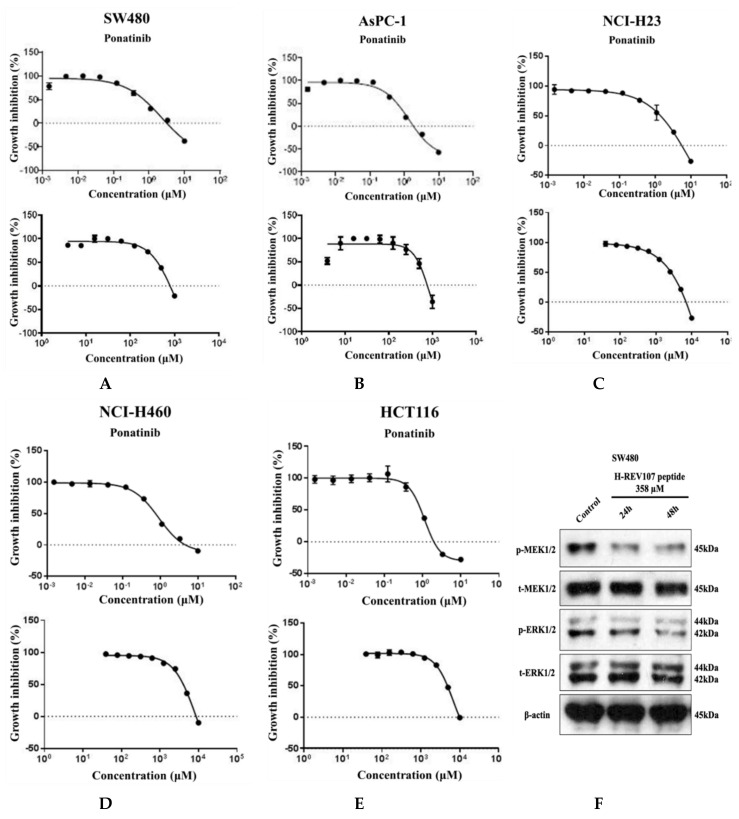

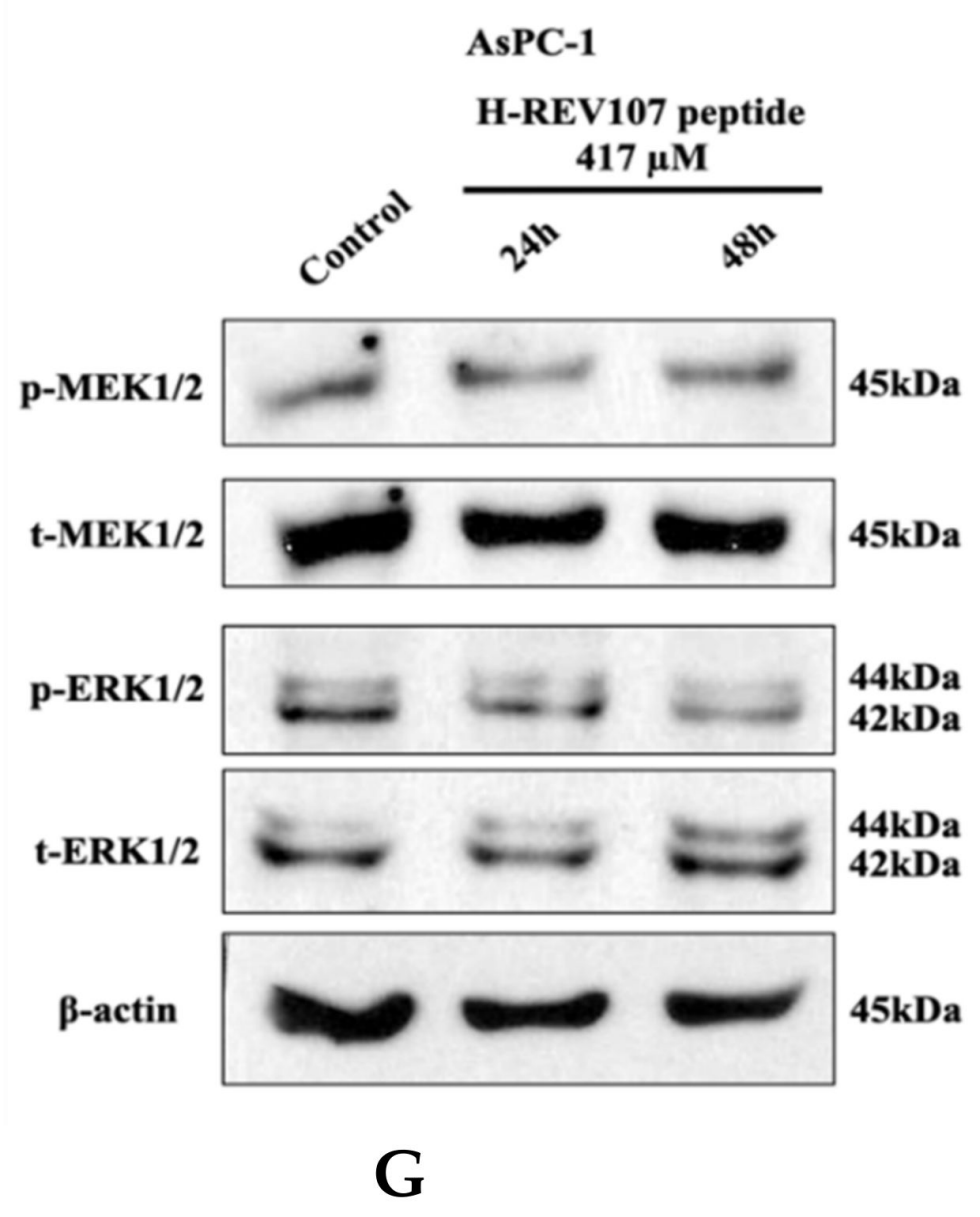
Cell proliferation assay and Western blot analysis. H-REV107 peptide was incubated for 72 h in five different cell lines, the (**A**) SW480, (**B**) AsPC-1, (**C**) NCI-H23, (**D**) NCI-H460, and (**E**) HCT116. Ponatinib was used for reference in five cell lines. Ponatinib to inhibit RAS-RAF-MAPK-ERK pathway was used to study cell proliferation in the KRAS mutant cancer cells for reference. The H-REV107 peptide was tested in triplicate and the data represent mean ± SD. (**F**,**G**) Western blot analysis of phosphorylated (*p*)-MEK1/2, total (*t*)-MEK1/2, *p*-ERK1/2, and t-ERK is shown in SW480 and AsPC-1 cells incubated with H-REV107 peptide for 24 and 48 h. Equal loading is shown by ß-actin.

Five tumor cell lines of different lineages were used to test the inhibitory effect of H-REV107 peptide on their proliferations. To evaluate the inhibitory effect of this peptide on cells that were in proliferative state, a longer incubation period was also tested. When the peptides were incubated with cells for up to 72 h, the decrease in cell viability was observed on SW480 (colon cancer), AsPC-1 (pancreatic cancer), NCI-H23 (lung cancer), NCI-H460 (lung cancer), and HCT116 (colon cancer) (Figure 3). H-REV107 peptide exhibited a GI_50_ value of 358 μM against SW480 cell line while it showed a GI_50_ value of 417 μM against AsPC-1 cell line. In addition, H-REV107 peptide also displayed antitumor activities with GI_50_ values in millimolar ranges for other tumor cell lines (NCI-H23, NCI-H460, and HCT-116). 

MTT results showed that higher concentration state of H-REV107 peptide had an effect on the proliferation of wild-type KRAS A549 cell line than other tumor cell lines. On the basis of the inhibitory effects of H-REV107 peptide on tumor cell proliferation, we examined H-REV107 peptide impact on the MEK/ERK signaling pathway in SW480 and AsPC-1 cells. We observed that H-REV107 peptide could decrease the phosphorylation levels of MEK/ERK in SW480 cell line, but the tendency to decrease of the phosphorylation levels of MEK/ERK in the AsPC-1 cell line was little. Western blotting had little effect on the expression level of the total MEK/ERK protein (Figure 3F,G).

### 2.4. H-REV107 Peptide Can Suppress Pancreatic Tumor Growth

To investigate the anti-tumor effect of H-REV107 peptide in vivo, we inoculated pancreatic AsPC-1 cancer cells on the skin in a xenograft nude mouse model and injected the peptide into the abdominal cavity.

**Figure 4:**
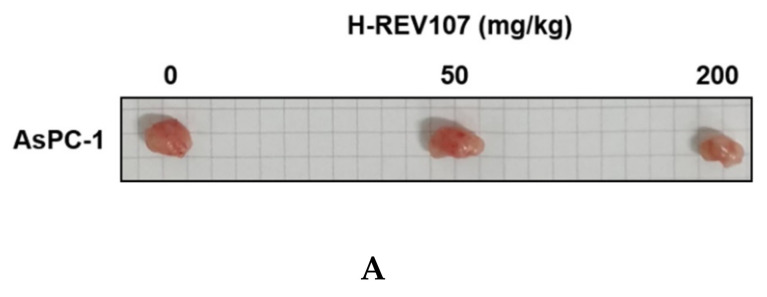

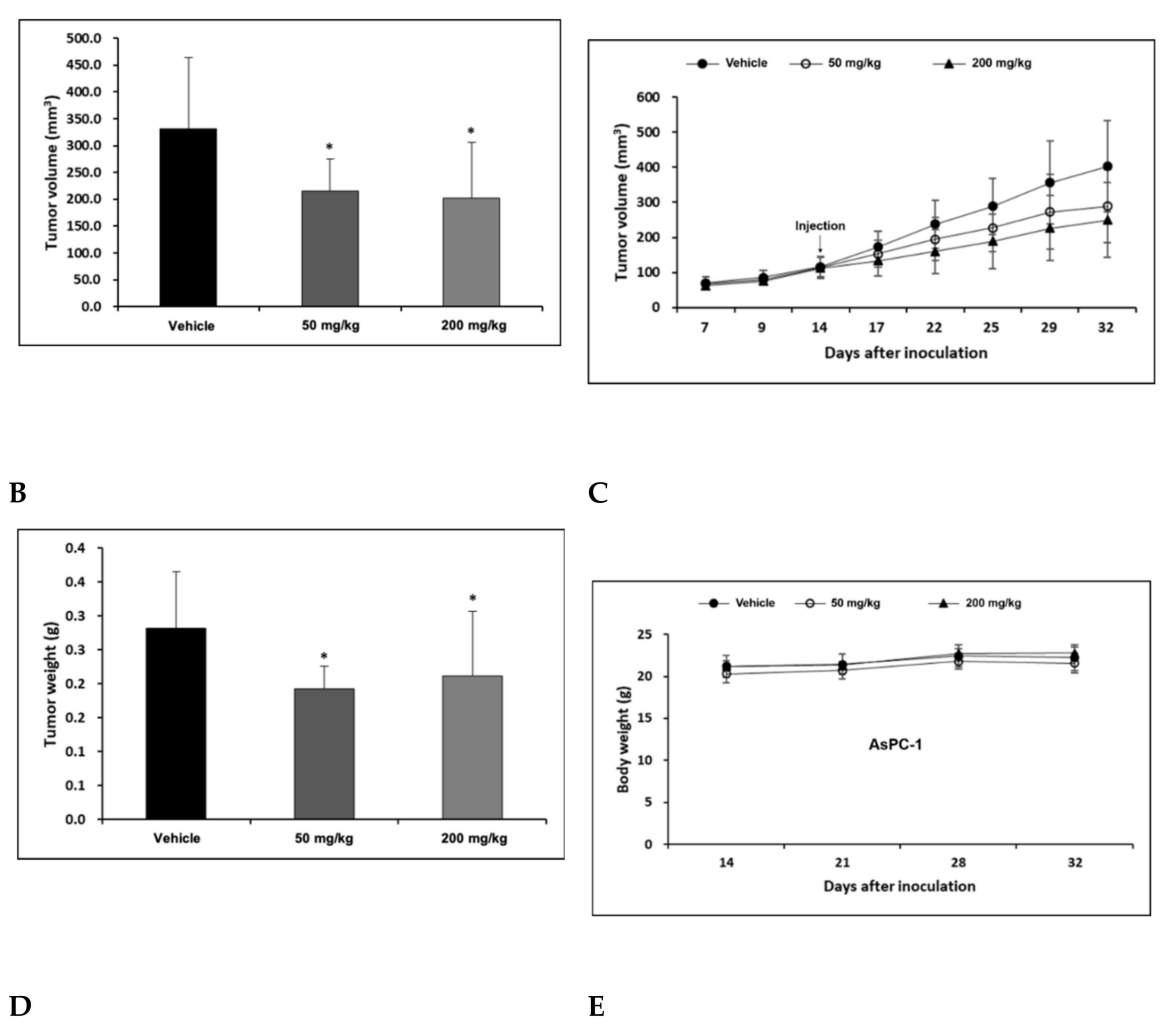
Suppression of tumor growth by H-REV107 peptide in mice inoculated with pancreatic AsPC-1 cancer cells. AsPC-1 cells were inoculated on the skin in a xenograft mouse model and injected the H-REV107 peptide into the abdominal cavity on a daily basis. (**A**) Photograph of tumor treated with the indicated dose of H-REV107 is shown. (**B**–**C**) Volume and weight of tumor were measured. Data are presented as means ± SD. Statistic tests were performed using Student’s *t*-test and Dunnett’s test. The minimum level of statistical significance was set at a *p*-value of 0.05 for all the analyses. (**D**,**E**) Body weight of each mouse was measured daily during injection of H-REV107 peptide.

AsPC-1 cell lines were cultured using the RPMI-1640 medium (10% FBS, 1% P/S), and the AsPC-1 cell lines were transplanted into the right flank skin with 1 × 10^6^/animal to produce a subcutaneous xenotransplantation animal model. The separation was conducted when the average volume of tumors grew to more than 100 mm^3^ after the transplantation of cancer cell lines, and consisted of three groups: Vehicle, H-REV107 peptide 50 mg/kg, and H-REV107 peptide 200 mg/kg. Five were deployed per group. The H-REV107 peptide was administered 15 times in the abdominal cavity for 3 weeks and weighed once a week during the test period and twice a week, and the tumor size was measured. During the experiment, no weight loss, infirmity, or behavioral abnormalities were observed and normal weight levels were displayed in all groups (Figure 4D,E). For tumor volume, from day 3 to day 32 after inoculation, the tumor volume was observed to decrease in all H-REV107 peptide doses compared to the Vehicle dose group, and the mean weight of the tumor measured during autopsy was 68% and 75% of the H-REV107 peptide 50 mg/kg and 200 mg/kg, respectively, compared with the Vehicle dose (Figure 4B,C). In this experiment, when combined with the above results, the H-REV107 peptide experimental group observed a lower growth rate of tumors compared to the Vehicle administration and the lowest growth rate in the group administered at a high concentration (200 mg/kg) (Figure 4A).

### 2.5. The Crystal Structure of KRAS G12V-H-REV107 Peptide Complex Was Determined

**Figure 5:**
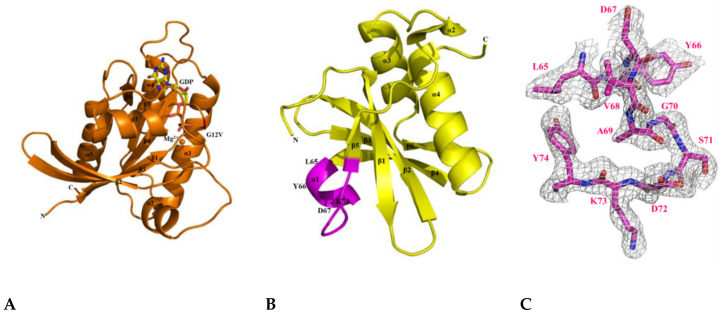

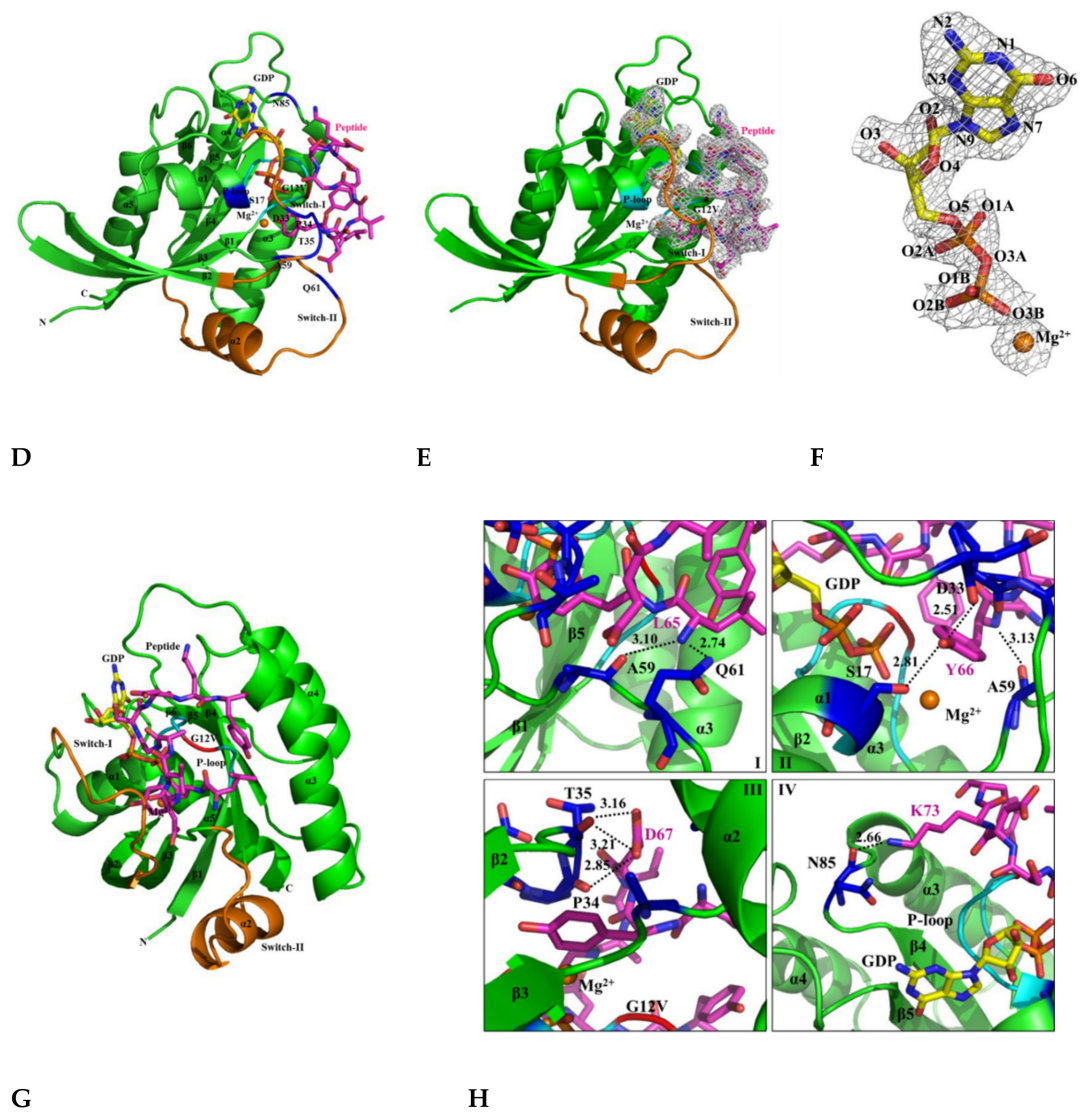
Crystal structures of the KRAS G12V, H-REV107, and KRAS G12V-H-REV107 peptide complex. (**A**) Ribbon representation of monomeric crystal structure of KRAS G12V with MgGDP (orange) is shown. Mg^2+^ ion is indicated by orange circle and the GDP is shown as yellow rod. MgGDP molecule is color-coded with C in gray, O in red, N in blue, P in purple, and Mg^2+^ ion in orange. (**B**) The crystal structure of the H-REV107 protein is shown in yellow and the fraction of the H-REV107 peptide is shown in purple. (**C**) The 2*F_o_-F_c_* electron density map of H-REV107 peptide was calculated at 2.3 Å resolution and contoured at 1σ (gray) at the peptide site. (**D**,**E**) Crystal structure of KRAS G12V-H-REV107 peptide complex is shown in green. Switch I and II regions in orange and P-loop in blue and peptide in purple are shown. (**F**) Electron density (2*F_o_-F_c_*) for bound MgGDP is contoured at 1σ (gray). (**G**,**H**) The binding regions of the KRAS and H-REV107 peptide are zoomed in on, and the hydrogen bonds in KRAS G12V and H-REV107 peptide complex are indicated by the black dashed lines.

After studying the molecular interaction of KRAS mutants and H-REV107, we attempted to make their crystals. First, we obtained the crystal of KRAS G12V (1–168)-MgGDP and determined its structure by the molecular replacement method using the dimeric structure of KRAS G12V with GDP (PDB ID: 5UQW) as a search model (Figure S2C) [25]. This structure contains the sequence of the full-length KRAS G12V-MgGDP protein, except for the last 20 residues in the hypervariable region (residues 169–188). The KRAS G12V (1–168)-MgGDP was obtained as a new monomeric crystal form that belongs to the hexagonal space group *P6_3_*, with unit cell parameters a = b = 82.547, c = 40.804 Å, α = β = 90, and γ = 120°. The crystallographic parameters and data collection statistics are summarized in Table 1. The structure of KRAS G12V-MgGDP consists of five α-helices (α1–α5) and six β-strands (β1–β6). The P-loop, and switch I and II regions in the structure are located in residues 10–17, 30–38, and 60–76, respectively (Figure 5A). The folding type of KRAS G12V-MgGDP is an α/β doubly wound shape, which is a mostly parallel sheet with helices on both sides. In this structure, KRAS G12V contains one molecule of MgGDP per asymmetric unit.

Next, the crystal structure of H-REV107 was determined by the molecular replacement method using the crystal structure of human HRASLS3 with a search model (PDB ID: 4DOT) [26]. We obtained the fraction crystal of the N-terminal domain (residues 1–125), but the electron density for residues 1–4 and 40–56 was poor (Figure S2D). The crystal belonged to the orthorhombic *P2_1_2_1_2_1_*, with unit cell parameters a = 42.935 Å, b = 52.997 Å, c = 62.796 Å, and α = β = γ = 90 ^o^ (Table S1). The structure of H-REV107 comprises four α-helices (α1–α4) and six β-strands (β1–β6) (Figure 5B). We designed a structural-based inhibitory peptide for targeting of KRAS mutants (Figure 5C). When the structures of our H-REV107 and HRASLS3 (PDB ID: 4DOT) proteins were superimposed over Cα atoms, the root mean square deviation (RMSD) showed a difference of 0.535 Å.

Finally, we successfully obtained the crystal of KRAS G12V-H-REV107 peptide (^65^LYDVAGSDKY^74^) complex and determined it at 2.3 Å resolution by the molecular replacement method using the coordinates of our KRAS G12V structure (Figure 5D and Figure S2E). The H-REV107 peptide was synthesized in a water-soluble form and its fraction was designed from α1 to β5 (amino acids 65–74). The H-REV107 peptide was bound to one molecule per four molecules of the KRAS G12V with a solvent content of 47% per asymmetric unit (Figure S2F). This structure comprises residues 1–168 of KRAS, 4 MgGDP, and 83 water molecules with an H-REV107 of 10-mer peptide. A ribbon representation of the crystal structure of the KRAS G12V-H-REV107 peptide complex was shown and the peptide and MgGDP electron density maps were clearly indicated (Figure 5E,F). The refinement is summarized in Table 1.

### 2.6. H-REV107 Peptide Was Strongly Docking in the Switch I and II Binding Pockets and the P-Loop of KRAS G12V

**Figure 6:**
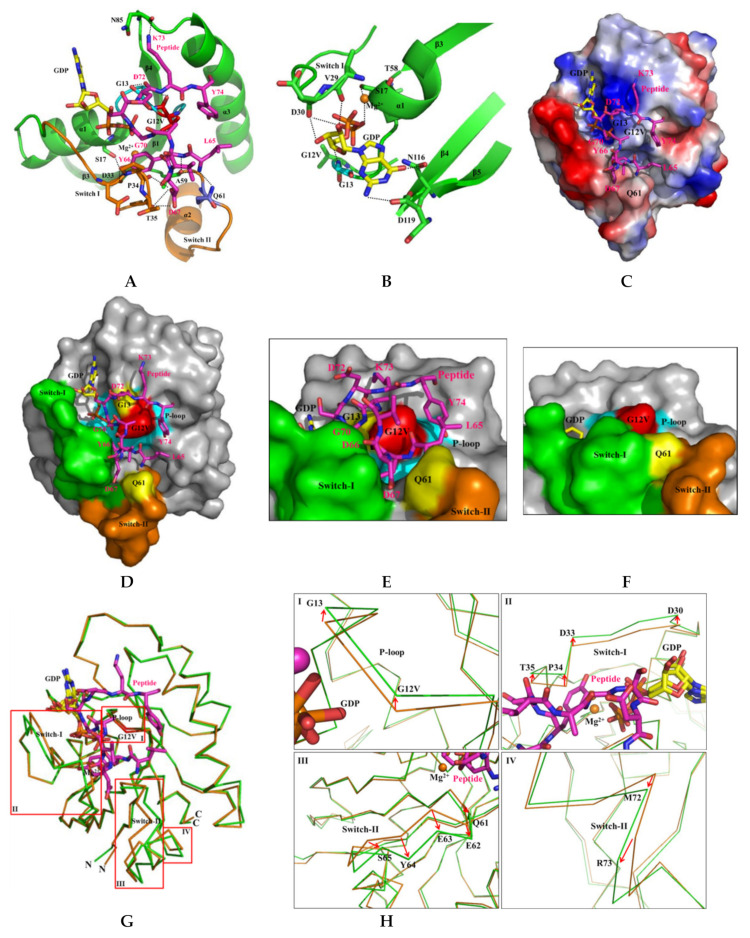
Detailed binding and structural characterization of KRAS G12V and H-REV107 peptide. (**A**) Binding region of the KRAS G12V and H-REV107 peptide is shown. The KRAS G12V, G13, and Q61 residues are coordinated and stabilized by L65, D72, K73, Y74, and MgGDP. The KRAS residues and H-REV107 peptide on the hydrogen-bonding network are labeled. (**B**) Interaction of the MgGDP and KRAS G12V-H-REV107 peptide is shown. MgGDP bound to residues of the P-loop, and switch I and II regions and NKXD motif of the KRAS G12V. (**C**) The relative distribution of the surface charge of the KRAS-H-REV107 is shown with the acidic region in red, the basic region in blue, and the neutral region in white. (**D**,**E**) Surface representations of the KRAS G12V-H-REV107 peptide complex are shown from the front and top sides. G12V (red), G13 and Q61 (yellow), switch I (green) and II (orange) regions, P-loop (blue), and peptide (purple) are shown. Peptide D67 deeply penetrated into the hole between the switch I and II pockets. (**F**) Surface representation of the KRAS G12V·MgGDP is shown. (**G**,**H**) Superposition of the Cα chain traces of the KRAS G12V (orange) and KRAS G12V-H-REV107 peptide (green) is shown. The boxed regions in the left panel are enlarged in the right panels, where the large conformational changes are drawn as arrow representations. The P-loop, and switch I and II regions of the KRAS G12V-H-REV107 peptide complex are more opened than those of KRAS G12V. Large conformational changes were observed in the switch I and II binding pocket regions of the KRAS G12V-H-REV107 complex from the structure of the KRAS G12V protein.

Notably, the H-REV107 peptide was strongly docking in close proximity to the switch I and II binding pockets and the near P-loop of KRAS G12V. Seven residues (L65, Y66, D67, G70, D72, K73, and Y74) of the H-REV107 peptide bound to the KRAS G12V protein (Figure 5G,H). The L65, D72, K73, and Y74 residues of the H-REV107 peptide interacted with KRAS G12V and G13, as well as with Q61. Additionally, KRAS G13 on the P-loop and H-REV107 peptide G70 strongly bound to β-phosphate of the GDP and make crucial contact with the phosphate of the GDP. The hydroxyl group of the H-REV107 peptide Y66 strongly bound to S17, D33, and A59 via hydrogen bonds, and interacted with the phosphate binding P-loop, and switch I and II regions. Charged H-REV107 peptide D67 also interacted with P34 and T35 in the switch I region, while charged H-REV107 peptide K73 interacted strongly with KRAS N85. Mg^2+^ bound to the OH groups of the S17 in the P-loop, T58 around switch II, and oxygen of the GDP β-phosphate (Figure 6A,B and Table 2). Proper metal coordination is crucial for tight nucleotide binding, with mutation of magnesium-coordinating residues leading to a preference for GDP over GTP [27,28]. Surprisingly, the H-REV107 peptide in the binding pocket surrounded and bound to KRAS mutations and the phosphate of GDP, and stabilized the KRAS mutants in an irreversible inactive GDP binding state. Specifically, it acted as an inhibitor of specific mutant KRAS targets (G12V, G12D, G12C, G13D, and Q61H). A surface representation of the KRAS–H-REV107 is shown in Figure 6C. The relative distribution of the surface charge is shown with the acidic region in red, the basic region in blue, and the neutral region in white. This peptide contains two negative charges of D67 and D72 and mainly binds to positively charged surfaces.

### 2.7. H-REV107 Peptide Stabilized the KRAS G12V in An Irreversible Inactive GDP Binding as An Open Conformation

When the structures of our KRAS G12V and KRAS G12V-H-REV107 peptide were superimposed on Cα atoms, the root mean square deviation (RMSD) showed a difference value of 0.503 Å (Figure 6G). Moreover, large root mean square deviations exceeding about 0.8 Å were observed on residues D30, E31, and D33-T35 in the switch I region; residues Q61-M67 and M72-T74 in the switch II region; and the V103-D108 surface loop region near α3 (Table 3). Large conformational changes of KRAS G12V were shown in the binding of H-REV107 peptide to KRAS G12V. Most large deviations were in the E63 and Y64 residues in the switch II region and affected peptide binding near Q61. The Q61 residue is essential for GTP hydrolysis and its mutation blocks Ras-mediated GTP hydrolysis and leads to tumor formation [16,17,18]. The structural differences between the KRAS G12V and KRAS G12V-H-REV107 peptide are mainly in the switch I and II regions. Interestingly, H-REV107 peptide binding can lead to rearrangement of the active sites (the switch I and II regions) of KRAS G12V. In this structure, the solvent accessible surface area of the KRAS G12V was found to be 18,587, while that of the H-REV107 peptide was 1975 Å^2^. Because the surface area of the KRAS G12V-H-REV107 peptide was 19,219 Å^2^, we can estimate that a large amount of the surface area (1343 Å^2^) was buried in the interface during the complex formation. Notably, the catalytic domain of KRAS around G12V on the P-loop region was surrounded by the H-REV107 peptide in the wide binding pocket, and D67 of the H-REV107 peptide penetrated deeply into the hole between the switch I and II pockets. In the H-REV107 peptide complex, the switch I region including P34 and T35 and the switch II region including Q61 were more open when compared to the form in the KRAS G12V MgGDP. Surface representations in the front and top of the KRAS G12V-H-REV107 peptide complex are shown in Figure 6C–F. When the H-REV107 peptide was bound, the switch I and II regions relaxed to a stereogenic form that no longer interacted with the active GTP nucleotide. The peptide wide binding pocket was located within the loop near the β2 (switch I), α2 (switch-II), and α3 helices of KRAS. Large conformational changes were observed in the switch I and II binding pocket regions of the KRAS G12V-H-REV107 complex from the structure of the KRAS G12V protein (Figure S4).

## 3. Discussion

The structure of KRAS G12V with the peptide in the binding pocket contained P-loop residues with the highly conserved G-domain sequence GXXXXGKS; residues V29, D30, and D33-T35 of switch I; and residues T58, A59, and Q61 near the DXXG motif (switch II), which is essential for interaction with the phosphate group of the GDP or Mg^2+^ ion [29]. Residues N116 and D119 of the conserved NKXD motif were also important for binding to the guanosine moiety of the GDP (Figure 6B). The peptide inhibitor bound to MgGDP and stabilized an inactive GDP-bound state. These findings indicate that H-REV10 peptide strongly and irreversibly binds to oncogenic KRAS G12V and fits well inside the binding pockets, leading to the opened rearrangement of its inactive sites (switch I and II) and blockage of the association of KRAS mutants with GTP. Finally, it can successfully inhibit the activity of oncogenic KRAS mutants; however, because it is non-toxic, this peptide leaves the normal protein intact.

In our structure, inhibitor H-REV107 peptide was covalently bonded to seven residues (S17, D33, P34, T35, A59, Q61, and N85) of the KRAS. In addition, two residues (G12V and G13) interacted with the H-REV107 peptide. Five residues (G13, V29, D30, N116, and D119) of the KRAS were bonded to GDP, and H-REV107 peptide was also bonded to GDP (Table 2). The structure of the KRAS G12V-H-REV107 peptide was found to be an opened conformation with an inactive GDP-bound state, and large conformational changes were shown at the regions of switch I (T35) and switch II loops (aa 60–74).

Characterizing the molecular details describing how H-REV107 binds KRAS G12V is essential to understanding the molecular mechanism through which H-REV107 inhibits oncogenic KRAS mutants. It is known that the H-REV107 protein family can inhibit the RAS signaling pathway, but the molecular mechanism responsible for this is unknown. In this study, we investigated the direct interaction between KRAS and H-REV107 protein/peptide. In surface plasmon resonance (SPR), immobilized KRAS mutants (G12V and G12D) and H-REV107 protein/peptide showed strong binding affinity. Guanine nucleotide binding was regulated by the H-REV107 peptide, and the binding of H-REV107 peptide to KRAS mutants showed decreased GTP binding affinity of KRAS mutants. Notably, both KRAS G12V and G12D were sensitive to H-REV107 peptide, while H-REV107 peptide blocked GTP binding to the KRAS mutants. Treatment with the H-REV107 peptide effectively inhibited pancreatic cancer and colon cancer cell lines in cell proliferation assay by inducing apoptosis. Compared to small inhibitor compounds, the working concentration of cellular activity of peptides is usually higher. We plan to further experiment by modifying the peptide in the future in order to improve the potency, and to include control peptides that have reduced RAS binding. Moreover, we found that the H-REV107 peptide downregulated the phosphorylation of the MEK/ERK signaling pathways. Specially, the H-REV107 peptide suppressed pancreatic tumor growth by reduction of tumor volume and weight in xenotransplantation mouse models.

On the basis of the information obtained from these studies, we investigated the structural aspects of KRAS G12V and H-REV107 interaction and determined the crystal structure of H-REV107 peptide-bound KRAS G12V in the MgGDP state at a resolution of 2.3 Å. In the present study, the H-REV107 peptide was strongly docking in close proximity to the switch I and II binding pockets, and near the P-loop of KRAS G12V. Seven residues (L65-D67, G70, and D72-Y74) of the H-REV107 peptide bound to KRAS G12V and four residues (L65 and D72-Y74) of the H-REV107 peptide interacted with KRAS G12V, G13, and Q61. The H-REV107 peptide interacted with the residues on the P-loop (G12, G13, and S17), switch I (D33, P34, and T35), switch II (A59 and Q61) and N85 of KRAS G12V. In addition, hydroxyl groups of residues S17 and T58 interacted with Mg^2+^ ion and residues G13, V29, and D30 of the P-loop, while the switch I regions interacted with N116 and D119 of the conserved NKXD motif and were found to be important for binding to the guanosine moiety of the GDP (Figure 6B–H).

As indicated by the structures of the KRAS G12V and H-REV107 peptide complex, the binding of H-REV107 peptide moves the switch I and II regions even further away and interferes with GTP binding itself. On the basis of these considerations, a function of the H-REV107 peptide is to attenuate KRAS signaling by blocking the GTP binding. The currently available studies suggest that solving the outstanding issues regarding KRAS could lead to development of effective drugs that have a significant impact on cancer treatment [30]. Our data provide detailed information regarding the molecular mechanism responsible for KRAS and H-REV107 interaction that improve our understanding of the biological activity of oncogenic KRAS mutants and may lead to development of a novel KRAS inhibitor. Future research will explore other KRAS mutations and inhibitors of cancer development.

## 4. Materials and Methods

### 4.1. Recombinant Protein Expression and Purification

His-tagged human KRAS wild-type and mutants (G12V, G12D, G12C, G13D, and Q61H) and H-REV107 were transformed into *Escherichia coli* BL21 (DE3) cells. Each individual colony was inoculated into 5 L of Luria-Bertani (LB) medium enriched with 10 μg/mL kanamycin, after which the bacteria were grown for 16 h at 37 °C. These cells were then added to 2 L of LB containing antibiotics and grown at 37 °C until the OD_600_ reached 0.5–0.6. The expression of the proteins was induced by 0.5 mM isopropyl-thio-β-D-1-thiogalactopyranoside (IPTG) at 25 °C, after which the bacterial cells were harvested by centrifugation at 3830 × *g* for 25 min at 4 °C, then disrupted by sonication in lysis buffer (50 mM HEPES (pH 7.5), 100 mM NaCl, and 2 mM MgCl_2_). The supernatant was subsequently incubated with Ni-NTA resin (BioRad) for 10 min. After washing, the bound protein was eluted from the beads with elution buffer (50 mM HEPES (pH 7.5), 500 mM NaCl, 2 mM MgCl_2_, and 400 mM imidazole). The eluted protein was further purified by fast protein liquid chromatography (FPLC) using a Superdex 200 10/300 GL column equilibrated with 50 mM HEPES (pH 7.5), 100 mM NaCl, and 2 mM MgCl_2_. The purity and identity of the proteins were determined by 15% SDS-PAGE. All mutagenesis experiments were conducted using the QuickChange method.

The cDNA fragment for the N-terminal domain of H-REV107 (residues 1–125) was amplified by PCR and cloned into pGEX-4T-1 vector (in frame with N-terminal GST tag). The respective plasmid was then transformed into *E. coli* BL21 (DE3) cells and grown to an optical density (OD) of 0.5 in LB medium with 50 μg/mL ampicillin. Next, 0.5 mM IPTG was added to the culture and protein was overexpressed for 16 h at 25 °C. Cells were subsequently lysed in 1× PBS buffer (4.3 mM Na_2_HPO_4_, 1.47 mM KH_2_PO_4_, 137 mM NaCl, and 2.7 mM KCl (pH 7.4)). The clear GST-H-REV107 supernatant was then loaded onto a Glutathione-Sepharose 4 Fast Flow column (GE healthcare) at a flow rate of 0.5 mL/min, after which the column was washed extensively using 30 mL of 1× PBS. The bound proteins were subsequently eluted in buffer (50 mM Tris-HCl (pH 8.0), 200 mM NaCl, and 5 mM glutathione). Finally, gel filtration was performed by FPLC using a Superdex 200 10/300 GL column.

### 4.2. Size-Exclusion Chromatography–Multi-Angle Light Scattering (SEC-MALS)

KRAS G12V was mixed in a 1:1 molar ratio with purified H-REV107 protein (1–125). The bound mixture was then applied onto a size-exclusion chromatography column TSK-gel-G3000SW_XL_ (Tosoh) connected to a DAWN HELEOS II multi-angle light scattering detector (WYATT Technology). The column was subsequently equilibrated with buffer (50 mM HEPES (pH 7.5), 100 mM NaCl, and 2 mM MgCl_2_) until the baseline of the MALS detector was stable. The run was performed using 2 mg/mL of sample applied at a flow rate of 0.5 mL/min.

### 4.3. Peptide Synthesis

The peptide (^65^LYDVAGSDKY^74^) of H-REV107 was designed on the basis of interaction modeling between KRAS and H-REV107 proteins. The peptide was produced using Fomc solid-phase peptide synthesis (SPPS) (Peptron Inc., Korea) and purified by reverse-phase high performance liquid chromatography (RP-HPLC) to >95% purity. Finally, the peptide was identified using liquid chromatography/mass spectrometry (LC/MS; HP 1100 Series; Agilent Technology, Santa Clara, CA, USA).

### 4.4. Biacore Biosensor Analysis

Measurement of the apparent dissociation constant (K_D_) between H-REV107 protein/peptide and KRAS mutants (G12D and G12V) was conducted using a Biacore T100 biosensor (GE Healthcare, Sweden). To accomplish this, each mutant KRAS protein in 10 mM sodium acetate (pH 5.0) was coupled to a CM5 sensor chip (GE Healthcare) at a concentration corresponding to 2300 response units (RU) using an amine coupling method. A flow path including two cells was then used to concurrently measure the kinetic parameters from one flow cell containing the mutant KRAS-immobilized sensor chip to another flow cell containing an underivatized chip. For kinetic measurement at room temperature, H-REV107 protein/peptide mixtures at concentrations ranging from 1.5 to 200 μM were set up by dilution in HBS buffer (150 mM NaCl, 3 mM EDTA, 10 mM HEPES, and 0.005% surfactant P20; pH 7.4). Each sample was subsequently injected into the flow cell at a rate 10 μL/min, after which the immobilized ligand was regenerated by injecting 50 mM NaOH.

### 4.5. Auto-Isothermal Titration Calorimetry (Auto-ITC)

The dissociation constant and stoichiometry between His-tagged KRAS mutants (G12V, G12D, G12C, G13D, and Q61H) and H-REV107 protein/peptide were determined from auto-isothermal titration calorimetry measurements. The proteins were dialyzed in buffer (50 mM HEPES (pH 7.5), 100 mM NaCl, and 2 mM MgCl_2_) at a concentration of 0.1 mM. H-REV107 protein/peptide were solubilized in the same buffer at a concentration of 1.0 mM. Titrations measurements that consisted of 20 injections with 200 s spacing were performed at 25 °C while the syringe was stirred at 1000 rpm. The determined K and ΔH values were used to calculate ΔS from the standard thermodynamic equation. Auto-ITC experiments were performed using the MicroCal AutoITC200 (GE Healthcare, Sweden) and the data were analyzed using Origin 7.0 program.

### 4.6. Circular Dichroism (CD) Spectrometer Analysis 

Circular dichroism (CD) spectrometry was used to estimate the secondary structure of the proteins [31]. Samples were analyzed using a J-1500 Spectrometer (JASCO Inc, USA) with a 1 mm pathlength cell over the 190–260 nm range (Far UV). The concentrations of recombinant wild-type KRAS or mutant KRAS (G12V, G12D, G12C, G13D, and Q61H) proteins were 0.5 mg/mL in 50 mM HEPES (pH 7.5), 100 mM NaCl, and 2 mM MgCl_2_. The CD spectra of the native and irradiated proteins were acquired every 0.1 nm with a 1 s averaging time per point and a 1 nm band pass. Each spectrum was obtained as an average of three scans to reduce noise and smoothed before structure analysis was performed. Secondary structure prediction was performed using the JASCO Spectra Manager Version 2 CD Multivariate Secondary Structure Estimation (SSE) program.

### 4.7. Guanine Nucleotide Binding Assay

His-tagged KRAS mutants (G12V, G12D, G12C, G13D, and Q61H) were incubated in H-REV107 protein/peptide diluted into binding buffer (50 mM HEPES (pH 7.5), 100 mM NaCl, 2 mM MgCl_2_, 1 mM EDTA, and 1 mM DTT) at a 1:1 molar ratio, then applied to Ni-NTA resin and allowed to bind at 4 °C. In addition, the protein complex was incubated with [α-^32^P] GTP (2500 cpm/pmol) and GTP at 30 °C. To terminate the binding, ice-cold wash buffer (20 mM Tris-HCl (pH 7.4), 100 mM NaCl, and 2 mM MgCl_2_) was added and elution of the bound protein was achieved using 200 mM imidazole. Protein-bound radioactive nucleotide was quantified by liquid scintillation counting.

### 4.8. Cell Proliferation Assay and Western Blot Analysis

Cell Counting Kit-8 (Dojindo, Japan) was used to measure the inhibition effect of H-REV107 peptide on the tumor cell proliferation. Cells (2 × 10^3^/well, 100 μL final volume per well) for HCT116 and NCI-H460, 5 × 10^3^ cells/well for SW480 and NCI-H23, and 1 × 10^4^ cells/well for AsPC-1 cell lines were used in this assay (Table 4). All cells were allowed to attach for 24 h after plating, and the cells were treated with fresh media the next day. The cells were then treated with H-REV107 peptide at increasing concentrations for 72 h. After the 72 h incubation, cell counting kit-8 (CCK-8) solution (10 μL/well) was added, and cells were incubated for a further 4 h at 37 °C CO_2_ incubator. The optical density (OD) of each well at 450 nm was measured by using the Microplate reader (BioTek, USA). Experiments were performed at least three times with representative data presented.

Cells were plated in a 100 mm cell culture dish and incubated under standard conditions (37 °C under a humidified atmosphere containing 5% CO_2_) in RPMI-1640 media with 10% fetal bovine serum, 100 μ/mL penicillin, and 100 μ/mL streptomycin (Welgene, Korea). After 24 h, the media was removed and replaced with fresh media (containing 0.1% DMSO) and then H-REV107 peptide was added to each dish. The treated H-REV107 peptide concentration was determined according to cell proliferation assay and GI_50_. After treatment of 24, 48, and 72 h, the cells were lysed using protein extraction solution PRO-PREPTM (iNtRON Biotechnology, Korea) containing a protease inhibitor cocktail for 30 min at 4 °C. After this, the cells were centrifuged at 15,115 x *g* for 30 min. Protein content in the supernatant was measured with Bradford protein assay using bovine serum albumin (BSA). The protein was separated by 10% SDS-PAGE and transferred onto an immobilon-P membrane (Millipore, USA). Membrane was blocked in ProNA Protein-free 5X general or phospho-block solution (TransLab, Korea) for 1 h at room temperature. The membrane was incubated with primary antibodies against phosphorylated (p)-MEK1/2 (Ser217/221), total (t)-MEK1/2, p-ERK1/2 (Thr202/Tyr204), and t-ERK1/2 (Cell Signaling Technology, USA) at 4 °C overnight, washed with 1X PBS containing 0.1% Tween (PBST) three times, and incubated with anti-IgG secondary antibodies (Santa Cruz Biotechnology, USA) in PBST for 1 h. The transferred protein band was visualized with EzWestLumi plus (ATTO corporation, Japan). Probing for ß-acitn was used as a loading control.

### 4.9. Tumor Xenograft Model

A total of 25 male five-week-old nude mice (weight: 14–17g) were purchased from Orient Bio Inc. The symptoms were observed once a day during the 7 day period. The weight was measured at the end of the purification period and the general symptoms were observed.

The cells used in the cancer animal model used human-specific pancreatic cancer cell liquor AsPC-1, which was defrosted more than 2 weeks before the animal model was created to obtain stable cell alcohol conditions, and were incubated three times. The growth medium used for cell culture was used by adding 10% fetal bovine serum and 1% penicillin/streptomycin to the RPMI-1640 medium used for AsPC-1 cell lines. On the day of the production of the cancer animal model, AsPC-1 cell line was removed from the cell culture flask by 0.25% Trypsin/EDTA and washed twice with PBS. The cell line was finally harried to HBSS + matrigel (1:1). Since the number of cells to be used in the experiment was 1 × 10^6^/head, the finally disheveled cell concentration was 1 × 10^7^/mL. It was stored in ice until the test was used. In the case of cell transplantation, 100 μL was transplanted. The separation was performed when the average volume of the tumor had grown to more than 100 mm^3^. A random separation of groups was conducted to ensure that the average tumor volume was equal in the day of separation, and the separation of groups was carried out into a total of three groups.

All animals were weighed and recorded once a week after transplanting cancer cells (weight unit: g). After transplanting cancer cell lines, the tumor size was measured twice a week on the basis of the start of administration. The tumor volume calculation method measured the longitude (L, length) and short diameter (W, width) and then applied the equation (L × W^2^)/2. All experimental procedures followed the Guidelines for the Care and Use of Laboratory Animals of the National Institutes of Health of Korea (Law No. 4379 on 31 May, 1991, Partial Amendment on 20 January, 2015, No. 12053), and were approved by the Institutional Animal Care and Use Committee (IACUC) of the Daegu Gyeongbuk Advanced Medical Industry Promotion Foundation, Republic of Korea (approval number: DGMIF18041702-01).

### 4.10. Crystallization, Data Collection, and Structure Determination

Crystals of the KRAS G12V were grown by hanging drop vapor diffusion at 20 °C for 2 weeks with polyethylene glycol 3350, 0.2 M potassium nitrate (pH 6.8), and acetonitrile in the well. The H-REV107 crystal was grown in a reservoir consisting of polyethylene glycol 3350 and 0.2 M potassium nitrate at pH 6.8. The crystal of the KRAS G12V-H-REV107 peptide complex was grown by soaking with polyethylene glycol 3350 and 0.2 M potassium nitrate (pH 6.8) and acetonitrile at 20 °C [32]. X-ray diffraction data of the KRAS G12V, H-REV107, or KRAS G12V-H-REV107 peptide complex were collected on the Pohang Light Source (PLS), beam-line 7A, Republic of Korea. The crystal was soaked in cryo-protectant solution containing additional glycerol and flash-frozen in liquid nitrogen for data collection under 100 K. Diffraction data were processed with the *HKL-2000* software [33] and the structure was solved by molecular replacement with *CCP4* [34]. The final model was produced by rounds of building in *COOT* [35], followed by refinement using *PHENIX* [36]. All structure figures were generated using the *PyMOL* program (http://pymol.org/2/). Final statistics of the collected data and refinement of the structures are shown in Table 1.

## 5. Conclusions

We demonstrated that H-REV107 peptide can suppress the KRAS activation function through the blocking of GTP binding to KRAS mutants. These results suggest that the blocking of GTP-binding by H-REV107 peptide could inhibit tumor cell proliferation through the downregulation of the KRAS pathway. Consequently, the reduction of GTP binding affinity of KRAS mutants by H-REV107 peptide in cancer cells influenced cell proliferation by inhibiting the RAS signaling pathway and inducing apoptosis. Our data will facilitate the development of novel drugs for inhibition of KRAS mutations.

## Figures and Tables

**Table 1:** Crystallographic statistics.

Data Collection	KRAS G12V- H-REV107 Peptide (PDB ID: 7C41)	KRAS G12V (PDB ID: 7C40)	H-REV107 (PDB ID: 7C3Z)
Space group	*P1*	*P6_3_*	*P2_1_2_1_2_1_*
Unit cell			
a, b, c (Å)	41.907, 84.339, 84.335	82.547, 82.547, 40.804	42.935, 52.996, 62.796
α, β, γ (^o^)	59.983, 89.961, 89.965	90.0, 90.0, 120.0	90.0, 90.0, 90.0
Wavelength (Å)	0.97934	0.97934	0.97934
Resolution (Å)	50.0–2.27	50.0–2.50	50.0–1.95
Completeness (%)	97.7 (81.6)	99.9 (100)	99.9 (100)
Observed reflections	328,760	823,903	367,550
Unique reflections	45,853	5543	10,814
*I/σ* (*I*)	16.5 (5.7)	27.2 (9.3)	36.9 (8.0)
R_merge_ (%) ^a^	10.2 (20.0)	12.8 (51.1)	8.8 (21.8)
Redundancy	3.9	21.6	14.0
R_cryst_/R_free_ (%) ^b^	26.03/28.34	18.46/24.24	23.43/26.29
Protein atoms	5348	1337	839
Solvent molecules	83	14	56
Mg^2+^/GDP molecules	4	1	0
Peptide	1	0	0
Bond length (Å)	0.009	0.009	0.008
Bond angle (^o^)	1.160	1.077	0.890
Average <B> factor (Å^2^) Peptide	31.3	0	0
Average <B> factor (Å^2^) Mg^2+^/GDP	19.3	24.4	0
Most favored regions (%)	97.2	94.0	97.0
Additional allowed regions (%)	2.2	6.0	3.0
Disallowed regions (%)	0.6	0	0

Values in parentheses are for the highest resolution shell. ^a^ R_merge_ = ∑|*I*_i_ *I*_m_|/∑*I*_i_, where *I*_i_ is the intensity of the measured reflection and *I*_m_ is the mean value of all symmetry-related reflections. ^b^ *R*_cryst_ = Σ||*F*_obs_| − |*F*_calc_||/Σ|*F*_obs_|, where *F*_obs_ and *F*_calc_ denotes the observed and calculated structure factor amplitude. *R*_free_ = ∑*_T_*||*F*_obs_| − |*F*_calc_||/Σ*_T_*|*F*_obs_|, where *T* is a test data set of about 5% of the total reflections randomly chosen and set aside prior to refinement.

**Table 2:** Interaction distances between KRAS G12V and H-REV107 peptide.

KRAS	H-REV107 Peptide	Interaction Distance (Å)	KRAS	H-REV107 Peptide	Interaction Distance (Å)
O (G12V)	N (K73)	4.04	O (N85)	NZ (K73)	2.66
N (G12V)	O (Y74)	3.91	O2B (GDP)	O (G70)	2.50
N (G13)	O (D72)	3.89	N (KRAS G13)–O1B (GDP)		2.70
OG (S17)	OH (Y66)	2.81	O (KRAS V29)–O2 (GDP)		2.59
O (D33)	OH (Y66)	2.51	O (KRAS D30)–O2 (GDP)		2.91
O (P34)	OD1 (D67)	2.85	O (KRAS D30)–O3 (GDP)		2.76
O (T35)	OD1 (D67)	3.21	OD1 (KRAS N116)–O6 (GDP)		3.21
O (T35)	OD2 (D67)	3.16	OD2 (KRAS D119)–N2 (GDP)		3.14
O (A59)	N (L65)	3.10	OG (KRAS S17)–Mg^2+^		2.71
O (A59)	N (Y66)	3.13	O (KRAS T58)–Mg^2+^		3.03
NE2 (Q61)	N (L65)	2.74	O3B (GDP)–Mg^2+^		2.99

**Table 3:** Root mean square deviation (RMSD) values between KRAS G12V and KRAS G12V-H-REV107 peptide.

KRAS Residues	RMSD (Å)	KRAS Residues	RMSD (Å)
M1	1.83	E62	1.36
V12	0.48	E63	2.15
G13	0.48	Y64	2.31
D30	0.97	S65	1.58
E31	0.84	A66	1.26
Y32	0.78	M67	1.08
D33	0.92	M72	1.09
P34	1.37	R73	1.25
T35	1.03	T74	0.89
Q61	1.05	D108	0.99

**Table 4:** Characteristics of KRAS mutant cell lines.

Cell Lines	Tissues	KRAS Mutations
SW480	Colon	G12V
AsPC-1	Pancreas	G12D
NCI-H23	Lung	G12C
NCI-H460	Lung	Q61H
HCT116	Colon	G13D

## Supplementary Materials

The following are available online at https://www.mdpi.com/2072-6694/12/6/1412/s1, Figure S1: (A) The SDS-PAGE gel bands of the purified KRAS and H-REV107 proteins are shown. Lane 1, protein marker; lane 2, wild-type KRAS; lane 3, H-REV107, lane 4, KRAS G12V; lane 5, KRAS G12D; lane 6, KRAS G12C; lane 7, KRAS G13D, and lane 8, KRAS Q61H. (B) Circular dichroism (CD) of wild-type and mutant KRAS was used to investigate the stability of the secondary structure of KRAS in different types of point mutations. The CD spectra of the KRAS mutants showed that each mutation affected the conformation of KRAS to a different extent. The CD spectra were measured from 260 to 190 nm using a 0.05 pathlength cell, and CD signals were merged to CDNN. (C) The sequences and secondary structures of KRAS and H-REV107 are shown. The α-helices are shown as blue ellipses, β-sheets as orange arrows, and linker loops as gray lines. The G12V mutation of KRAS is shown as a red triangle. The binding residues of the KRAS to H-REV107 peptide are indicated with stars. Every 10 residues is indicated by a point, Figure S2. (A,B) SEC-MALS spectra of the KRAS and H-REV107 proteins are shown. The inset shows the value of the molecular weight of KRAS and H-REV107 determined from the MALS data analysis (black line: MALS, dashed line: molecular weight). (C-E) The crystals of the KRAS G12V, H-REV107, and KRAS G12V-H-REV107 peptide complex are shown. (F) The molecular packing of the KRAS G12V-H-REV107 peptide complex is shown. H-REV107 peptide was bound to one molecule per four molecules of the KRAS G12V per asymmetry unit, Figure S3. Isothermal titration calorimetry (ITC) analysis of the KRAS mutant and H-REV107 peptide/protein. The H-REV107 peptides or H-REV107 protein were titrated into the KRAS mutant solutions, and the measured K_D_ values are shown. (A) KRAS G12V with H-REV107 protein, (B) KRAS G13D with H-REV107 peptide, (C) KRAS Q61H and H-REV107 peptide, (D) KRAS G12C with H-REV107 peptide, Figure S4. (A) The Cα traces of the KRAS G12V (orange) and KRAS G12V-H-REV107 peptide (green) are superimposed and shown in stereoview. The switch I and II regions of the KRAS G12V-H-REV107 peptide were more opened than those of the KRAS G12V with MgGDP. (B-D) Surface representations of the front and top sides of the KRAS G12V-H-REV107 peptide complex are shown. (E) Surface representation of the KRAS G12V with MgGDP is shown.
