# Supplementary material for: A H-REV107 Peptide Inhibits Tumor Growth and Interacts Directly with Oncogenic KRAS Mutants
Source: Cancers (Basel). 2020 May 30;12(6):1412. doi: 10.3390/cancers12061412 (PMC7352977; doi:10.3390/cancers12061412)
Supplement: Supplementary file 1 [file cancers-12-01412-s001.pdf]

Supplementary material

A H-REV107 Peptide Inhibits Tumor Growth and Interacts Directly with Oncogenic KRAS Mutants

Chang Woo Han, Mi Suk Jeong, Sung Chul Ha and Se Bok Jang

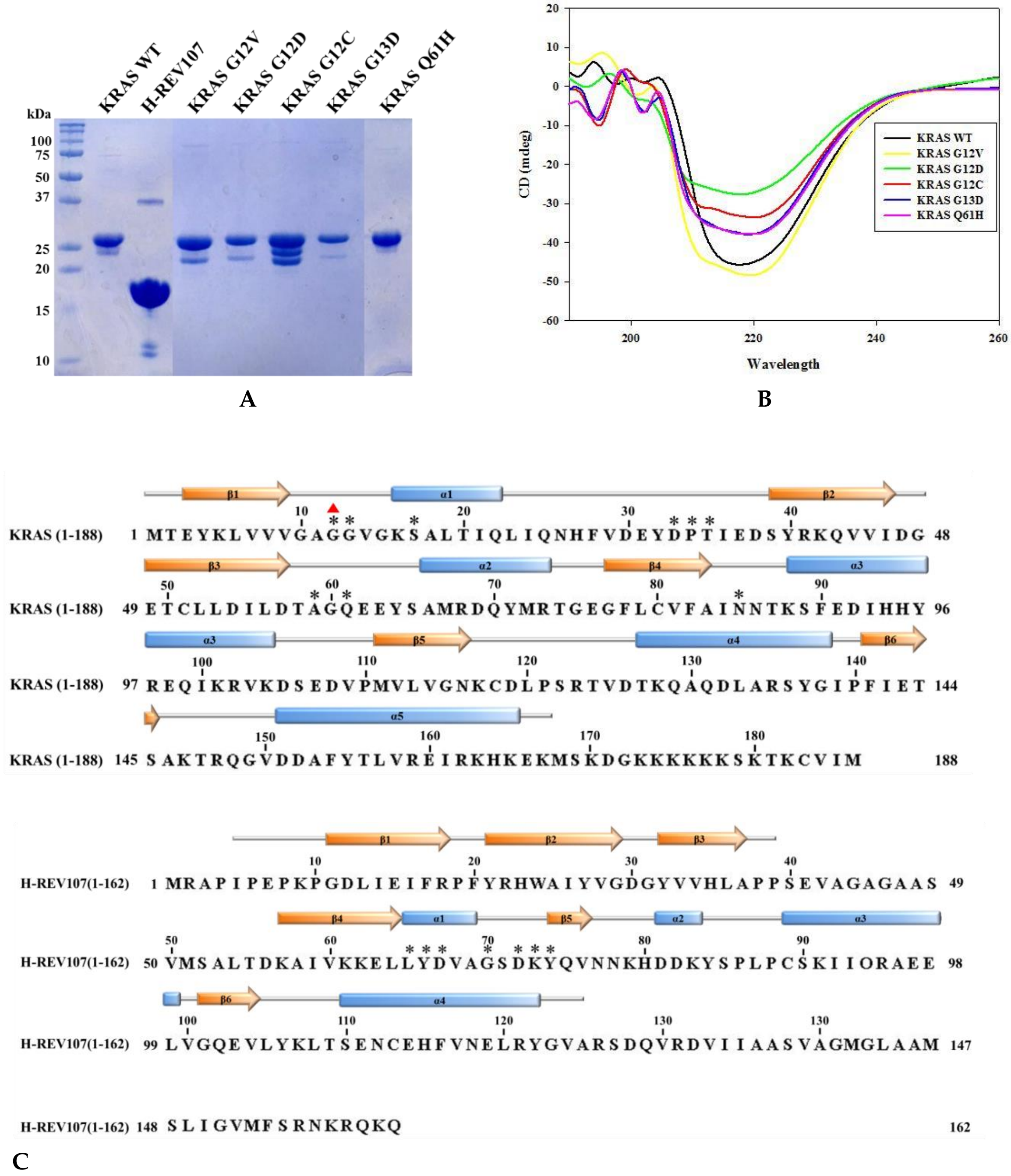

Figure S1. (A) The SDS-PAGE gel bands of the purified KRAS and H-REV107 proteins are shown. Lane 1, protein marker; lane 2, wild-type KRAS; lane 3, H-REV107, lane 4, KRAS G12V; lane 5, KRAS G12D; lane 6, KRAS G12C; lane 7, KRAS G13D, and lane 8, KRAS Q61H. (B) Circular dichroism (CD) of wild-type and mutant KRAS was used to investigate the stability of the secondary structure of KRAS in different types of point mutations. The CD spectra of the KRAS mutants showed that each

mutation affected the conformation of KRAS to a different extent. The CD spectra were measured from 260 to 190 nm using a 0.05 pathlength cell and CD signals were merged to CDNN. (C) The sequences and secondary structures of KRAS and H-REV107 are shown. The α-helices are shown as blue ellipses, β-sheets as orange arrows and linker loops as gray lines. The G12V mutation of KRAS is shown as a red triangle. The binding residues of the KRAS to H-REV107 peptide are indicated with stars. Every 10 residues is indicated by a point.

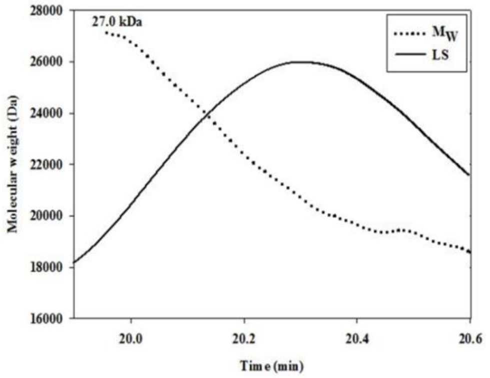

A

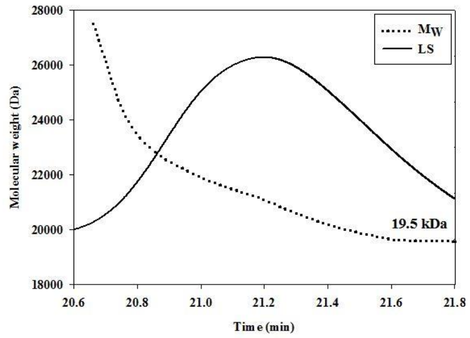

B

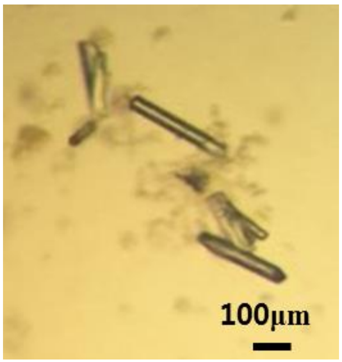

C

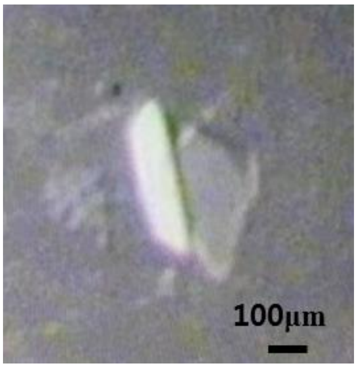

D

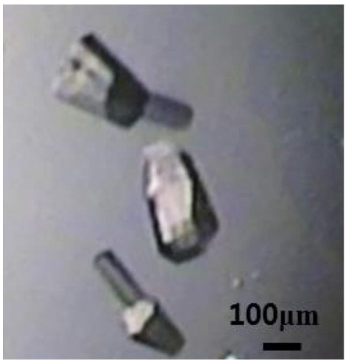

E

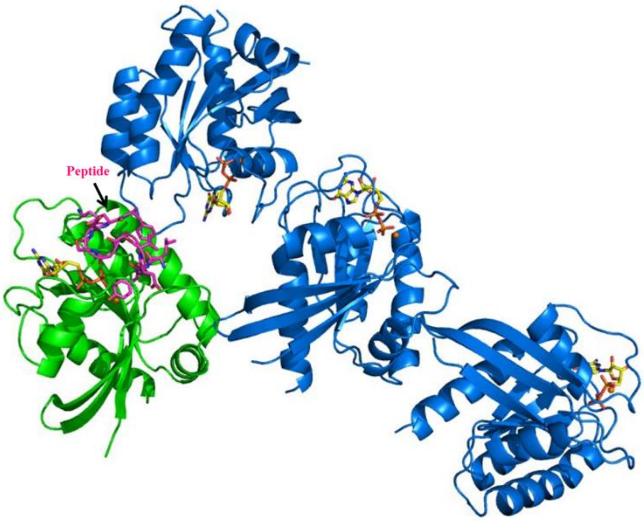

F

Figure S2. (A-B) SEC-MALS spectra of the KRAS and H-REV107 proteins are shown. The inset shows the value of the molecular weight of KRAS and H-REV107 determined from the MALS data analysis (black line: MALS, dashed line: molecular weight). (C-E) The crystals of the KRAS G12V, H-REV107 and KRAS G12V-H-REV107 peptide complex are shown. (F) The molecular packing of the KRAS G12V-H-REV107 peptide complex is shown. H-REV107 peptide was bound to one molecule per four molecules of the KRAS G12V per asymmetry unit.

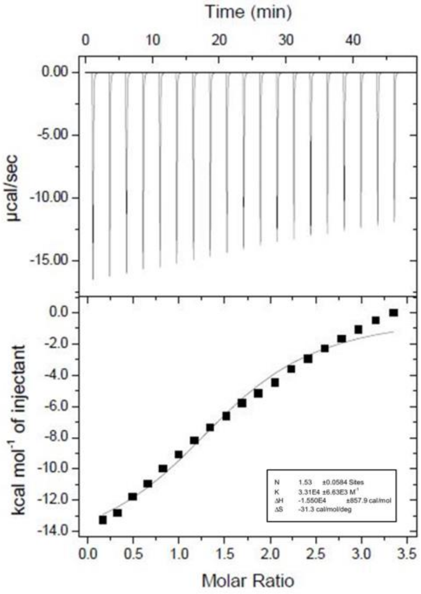

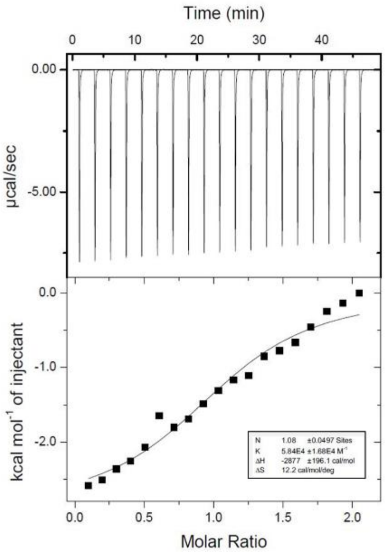

A **B**

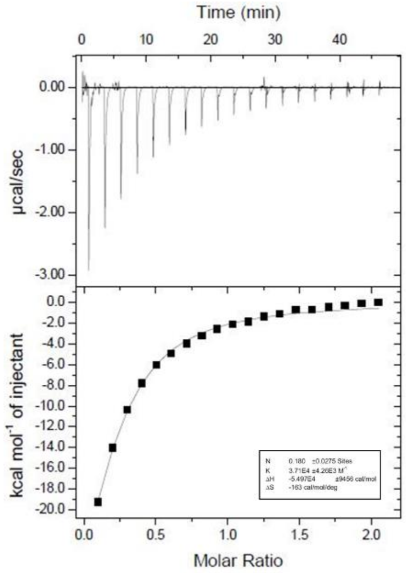

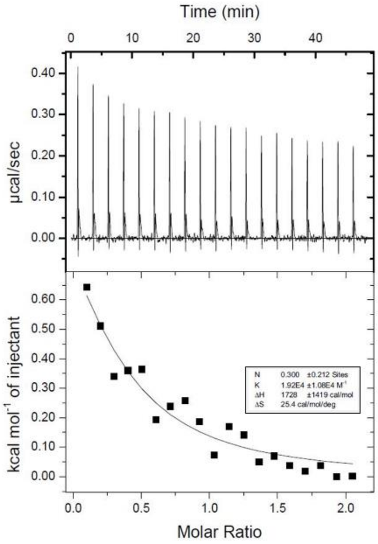

C **D**

Figure S3. ITC analysis of the KRAS mutant and H-REV107 peptide/protein. The H-REV107 peptides or H-REV107 protein were titrated into the KRAS mutant solutions, and the measured K_D values are shown. (A) KRAS G12V with H-REV107 protein (B) KRAS G13D with H-REV107 peptide (C) KRAS Q61H and H-REV107 peptide (D) KRAS G12C with H-REV107 peptide.

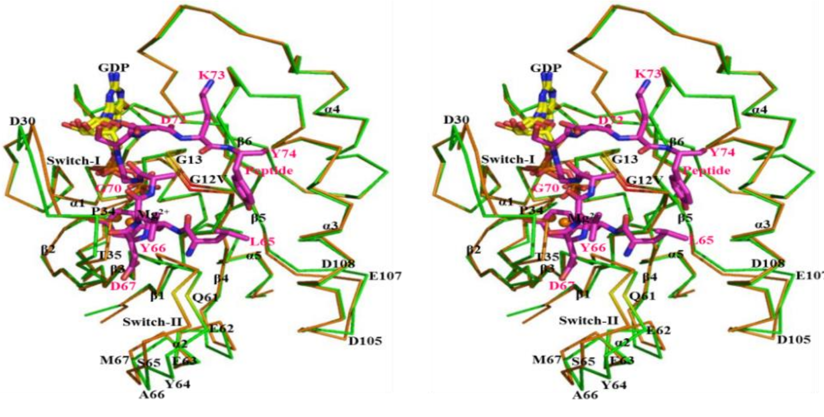

A

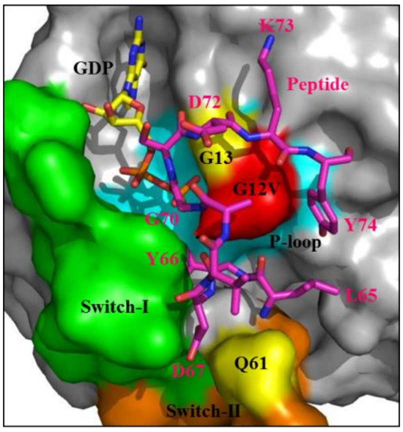

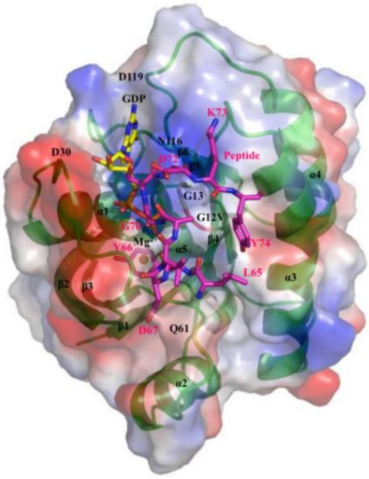

B **C**

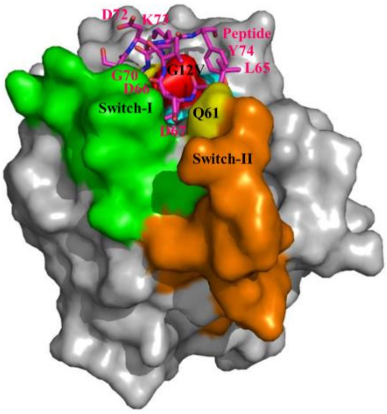

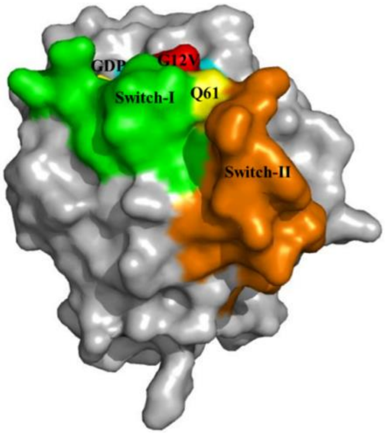

D **E**

Figure S4. (A) The Cα traces of the KRAS G12V (orange) and KRAS G12V-H-REV107 peptide (green) are superimposed and shown in stereoview. The switch I and II regions of the KRAS G12V-H-REV107 peptide are more opened than those of the KRAS G12V with MgGDP. (B-D) Surface representations of the front and top sides of the KRAS G12V-H-REV107 peptide complex are shown. (E) Surface representation of the KRAS G12V with MgGDP is shown.
